# Insertion of synthetic lesions on patient data: a method for evaluating clinical performance differences between PET systems

**DOI:** 10.1186/s40658-023-00610-2

**Published:** 2024-01-22

**Authors:** Quentin Maronnier, Nesrine Robaine, Léonor Chaltiel, Lawrence O. Dierickx, Thibaut Cassou-Mounat, Marie Terroir, Lavinia Vija, Delphine Vallot, Séverine Brillouet, Chloé Lamesa, Thomas Filleron, Olivier Caselles, Frédéric Courbon

**Affiliations:** 1 Nuclear Medicine Department, Institut Claudius Regaud, Institut Universitaire du Cancer Toulouse Oncopole, Toulouse, France; 2 Biostatistics Department, Institut Claudius Regaud, Institut Universitaire du Cancer Toulouse Oncopole, Toulouse, France; 3 Medical Physics Department, Institut Claudius Regaud, Institut Universitaire du Cancer Toulouse Oncopole, Toulouse, France; 4 Radiopharmacy Department, Institut Claudius Regaud, Institut Universitaire du Cancer Toulouse Oncopole, Toulouse, France

**Keywords:** Positron-emission tomography, Clinical, Performance, Methods, Simulation

## Abstract

**Background:**

Performance assessment of positron emission tomography (PET) scanners is crucial to guide clinical practice with efficiency. We have already introduced and experimentally evaluated a simulation method allowing the creation of a controlled ground truth for system performance assessment. In the current study, the goal was to validate the method using patient data and demonstrate its relevance to assess PET performances accuracy in clinical conditions.

**Methods:**

Twenty-four patients were recruited and sorted into two groups according to their body mass index (BMI). They were administered with a single dose of 2 MBq/kg ^18^F-FDG and scanned using clinical protocols consecutively on two PET systems: the Discovery-IQ (DIQ) and the Discovery-MI (DMI). For each BMI group, sixty synthetic lesions were dispatched in three subgroups and inserted at relevant anatomical locations. Insertion of synthetic lesions (ISL) was performed at the same location into the two consecutive exams. Two nuclear medicine physicians evaluated individually and blindly the images by qualitatively and semi-quantitatively reporting each detected lesion and agreed on a consensus. We assessed the inter-system detection rates of synthetic lesions and compared it to an initial estimate of at least 1.7 more targets detected on the DMI and the detection rates of natural lesions. We determined the inter-reader variability, evaluated according to the inter-observer agreement (IOA). Adequate inter-reader variability was found for IOA above 80%. Differences in standardized uptake value (SUV) metrics were also studied.

**Results:**

In the BMI ≤ 25 group, the relative true positive rate (RTPR) for synthetic and natural lesions was 1.79 and 1.83, respectively. In the BMI > 25 group, the RTPR for synthetic and natural lesions was 2.03 and 2.27, respectively. For each BMI group, the detection rate using ISL was consistent to our estimate and with the detection rate measured on natural lesions. IOA above 80% was verified for any scenario. SUV metrics showed a good agreement between synthetic and natural lesions.

**Conclusions:**

ISL proved relevant to evaluate performance differences between PET scanners. Using these synthetically modified clinical images, we can produce a controlled ground truth in a realistic anatomical model and exploit the potential of PET scanner for clinical purposes.

## Background

Positron emission tomography (PET) coupled with computed tomography (CT) is commonly used in oncology at various stages of the management of many cancers, either for diagnosis, treatment follow-up or monitoring [1]. However, the technical performance of PET limits the detection of lesions smaller than 10 mm in diameter, thereby impairing the detection and quantitation of the metabolic activity of small clusters of malignant tissue [2]. Various attempts at hardware or software improvements such as detection crystals, embedded electronics and reconstruction algorithms [3] are currently being studied to improve the overall performance of PET while mitigating the risk of overdiagnosis.

To assess PET performances, scientific experts from national and international authorities such as the National Electrical Manufacturers Association (NEMA) and the International Electrotechnical Commission (IEC) [4, 5] provide users and manufacturers with standard procedures. These procedures are relevant for investigating and benchmarking the scanners using test objects whose parameters are controlled [6] but are not appropriate for evaluating performances in clinical practice owing to patient complexity [7, 8]. Caution is therefore required when using these procedures to claim any improvement in diagnostic accuracy (i.e. sensitivity and specificity) in clinical practice. Procedures exist for standardizing the performance of PET scanners to facilitate multicentre studies, as recommended by the European Association of Nuclear Medicine Research 4Life (EANM/EARL) [9]. Both systems received EARL accreditation during the course of the clinical trial. EARL procedures validate results obtained from phantoms by evaluating new acquisition and reconstruction parameters for application in clinical practice. Nevertheless, patient examinations are constrained by the lack of ground truth associated with their clinical data.

The gold standard method to determine lesion-based diagnostic accuracy expressed as the true positive and true negative rates from patient examinations is based on invasive pathological sampling or a long follow-up [10]. For non-specific PET tracers such as ^18^F-fluorodeoxyglucose (^18^F-FDG), it is even more difficult to transpose these parameters without a pathological gold standard [11]. Enhanced detection sensitivity may lead to a higher risk of suspicious findings, potentially resulting in false positive cases.

Our team previously described and evaluated a method combining physical data and insertion of synthetic lesions (ISL) under specific experimental conditions [12]. The method produced equivalent visual and semi-quantitative results even in challenging situations such as sub-centimetric and low-contrast targets.

Studies coupling such simulation with clinical data have already been published. Several aspects of PET imaging are addressed, including reconstruction algorithms, corrections, detection and semi-quantitation. A team developed a lesion insertion tool for PET/MR to evaluate the semi-quantitative accuracy of various PET attenuation correction approaches [13]. In another study, Wangerin et. al. [14] inserted synthetic lesions into patient data to evaluate and compare lesion detectability between two reconstruction algorithms and two anatomical locations. Gabrani-Hanif et. al. [15] also used the same procedure in human perception studies to assess the limits of lesion detection for various lesion sizes and contrasts. All these studies demonstrate the contribution of ISL and highlight opportunities for evaluating medical imaging devices and image processing techniques [16].

In clinical studies, the method uses data from real anatomical models, thereby approaching clinical practice with more accuracy. Moreover, simulation can both target and control synthetic data, providing a reliable alternative to conventional invasive methods for establishing the ground truth on clinical data.

The goal of the current study is to use ISL in a clinical study to establish the detection rate of two PET exams from the same patient and therefore evaluate the relevance of using ISL to compare clinical performances of PET systems.

## Methods

### PET/CT systems

Two different PET/CT scanners were used, the Discovery-IQ (DIQ) and the Discovery-MI (DMI) (General Electric Healthcare, Chicago, IL, USA), both with a 5-ring configuration leading to an equivalent axial field of view (FOV). The technical specifications of the devices are detailed in Table 1. Considering the size and composition of the crystals in each system, we expect to observe differences in terms of detection limits and semi-quantification. The size of the crystals is a very important design parameter, as it has a direct impact on the spatial resolution of the PET systems. The DMI has detection crystals with thinner dimensions. In addition, the crystal used in the manufacture of the DMI is lutetium–yttrium-oxyorthosilicate (LYSO). Due to the properties of LYSO, it enables additional data correction using time of flight (TOF) information, resulting in an enhanced signal-to-noise ratio in the resulting PET images. These two factors suggest that the DMI should have the capability to detect more targets than the DIQ.

**Table 1:** Technical characteristics of DIQ and DMI PET/CT systems

	DIQ	DMI
Scintillator crystals	BGO	LYSO
Crystal size (mm^3^)	6.3 × 6.3 × 30	3.95 × 5.3 × 25
Light amplification	Analogic (PMT)	Digital (SiPM)
Time-of-flight (TOF)	No	Yes
Configuration (number of ring)	5-rings	5-rings
Axial/transverse field of view (FOV) (mm)	250/700	250/700

### Study design and participant

We designed a clinical study, IQversusMI (NCT03956459, May 20th, 2019), a monocentric prospective paired study designed to assess the relevance of ISL for evaluating PET performances from patient data. This study was conducted at the Institut Universitaire du Cancer Toulouse Oncopole (IUCT-O; Toulouse, France).

Eligible patient had cancer indication for ^18^F-FDG PET according to current clinical practice standards and were able to maintain a strict supine position on two occasions. They were ages > 18 years and had an Eastern Oncology Group Performance status (ECOG) of 0 or 1 and Karnofsky index > 70. The ECOG or Karnofsky Index assesses the overall health status of a patient. Conducting clinical research on patients in a compromised health state, particularly when there is no potential benefit for the patient, as is the situation in this study, is deemed unethical.

Key exclusion criteria were unbalanced diabetic patient and patients with a formal contraindication to certain imaging examinations (severe claustrophobia, heart valve, pacemaker, etc.). All patients provided written informed consent.

**Fig. 1:**
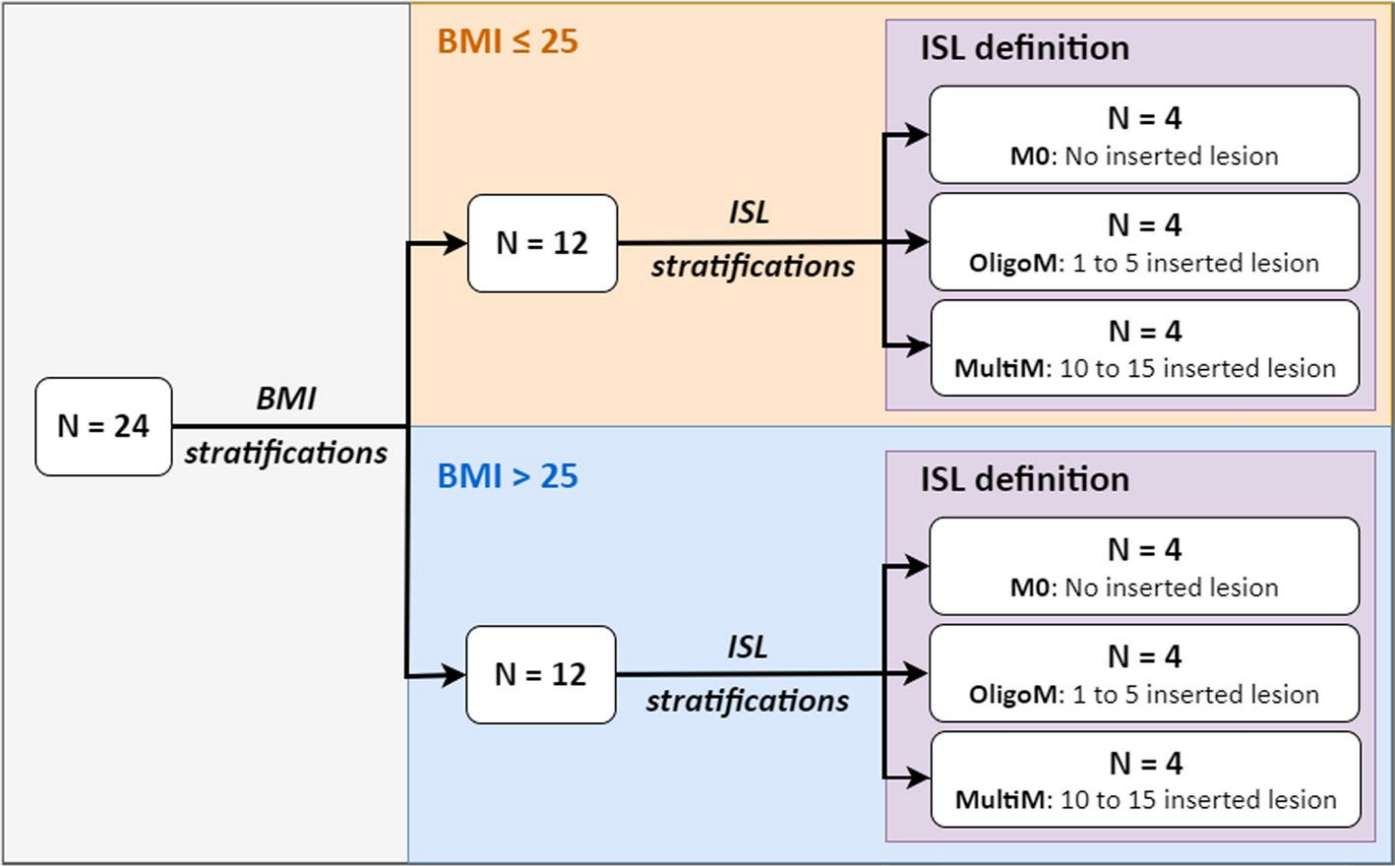
Stratification depending on BMI and tumour burden (i.e. ISL) of patients enrolled in the IQversusMI clinical trial

24 patients were stratified according to their body mass index (BMI) and the number of synthetic lesions to be inserted. Among the 24 patients enrolled, 12 had a BMI below or equal to 25 (BMI ≤ 25), while the other half were strictly above 25 (BMI > 25). In addition, both BMI cohorts were divided into three sub-cohorts in which the tumour burden varied. The detail of the stratification adopted for this clinical study is illustrated in Fig. 1.

Between July 2019 and February 2021, 24 patients were enrolled in this prospective monocentric study. In Table 2, we summarized demographic and baseline characteristics.

**Table 2:** Demographic and baseline characteristics of patients at inclusion

	BMI ≤ 25 (*N* = 12)	BMI > 25 (*N* = 12)
Age at inclusion (y.o.)—median [min; max]	63.0 [18; 81]	67.5 [47; 83]
Weight (kg)—median [min; max]	68 [58; 75]	84.5 [63.5; 110]
Height (cm)—median [min; max]	175 [163; 196]	171 [155; 185]
BMI (kg/m^2^)—median [min; max]	22.3 [16.7; 24.8]	26.6 [25.1; 40.4]

**Fig. 2:**
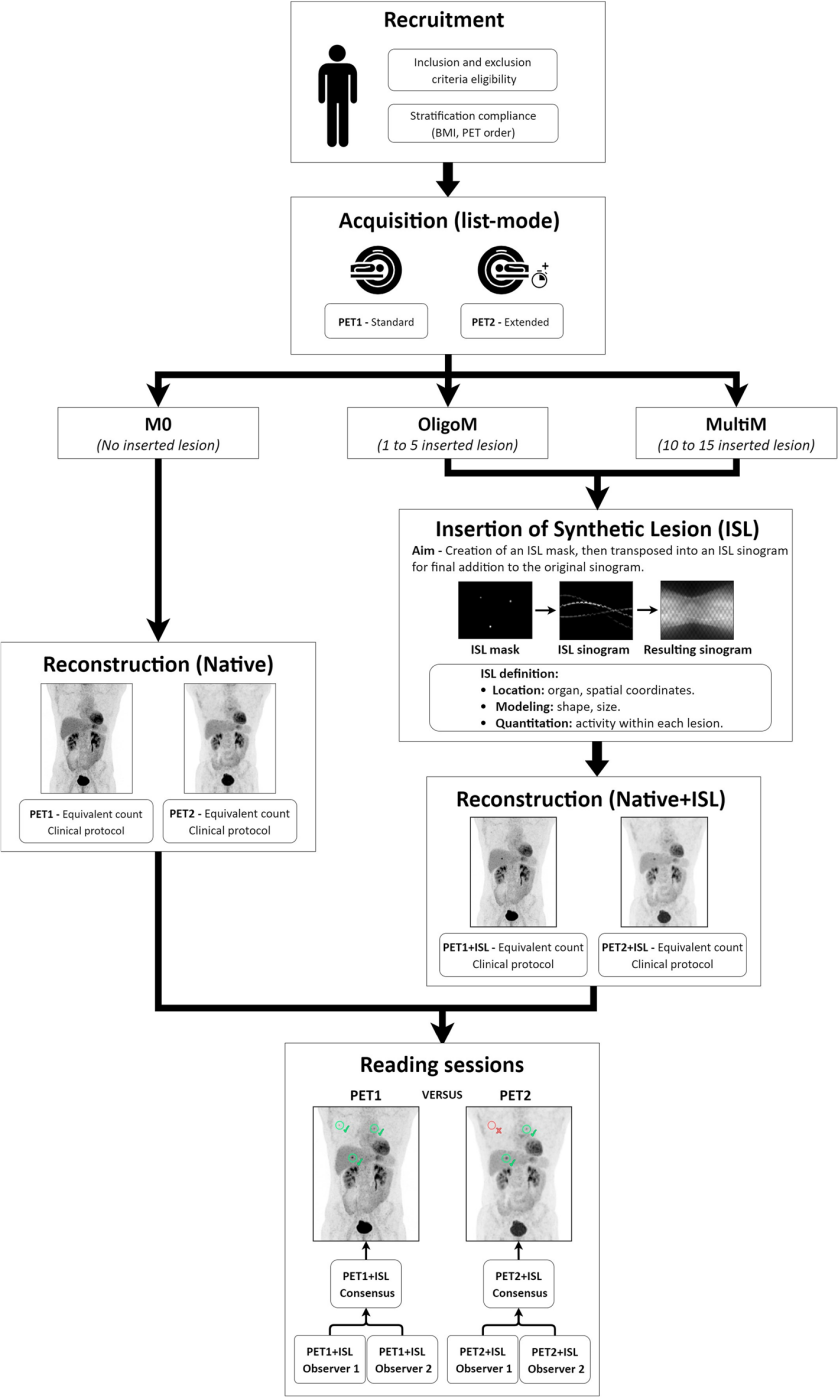
Entire workflow of IQversusMI clinical trial from patient recruitment to the completion of reading sessions by physicians. The figure illustrates an OligoM case with three inserted lesions

Patients underwent two consecutive PET/CT scans corresponding to different scanners, the DIQ and the DMI. The second examination was performed without additional injection of radiotracer and with a low-dose protocol CT. We defined anatomical locations identified on the consecutive exams of the same patient. Synthetic lesions were modelled and simulated on the PET images. An overview of the whole process is shown in Fig. 2.

### Acquisition and reconstruction parameters

Consecutive acquisitions were performed with an uptake time around 60 min and 85 min, respectively, after a single injection of ^18^F-FDG (2 MBq/kg). The order in which the examinations were performed was balanced in order to avoid the introduction of a bias due to the biodistribution of the radiotracer, which would differ at the time of each acquisition.

Acquisitions performed in list-mode were used to generate raw data with different durations in order to obtain similar counts per exam for each dataset. The second exam lasted longer to compensate for the radiotracer decay and to obtain equivalent statistics for both exams. These raw data were then reconstructed into PET images using Bayesian Penalized Likelihood (BPL) for the DIQ and with TOF additionally for the DMI. PET acquisition and reconstruction parameters are detailed in Table 3.

**Table 3:** PET acquisition and reconstruction parameters used with DIQ and DMI systems

PET acquisition and reconstruction parameters		
	DIQ	DMI
Radiotracer	^18^F-FDG	
Posology	2 MBq/kg	
Standard time per bed position	2 min/bed	
Matrix size	256 × 256	
Field of view (FOV)	600 mm	
Voxel dimension (*x*, *y*, *z*)	2.34 × 2.34 × 3.26 mm^3^	2.34 × 2.34 × 2.8 mm^3^
Algorithm	BPL	BPL
*β*	350	550
Correction	Attenuation, scatter, random, Point Spread Function (PSF)	Attenuation, scatter, random, Point Spread Function (PSF), TOF

CT technologies composing DIQ and DMI systems are very similar. In clinical practice, we are using the same CT protocols for both PET/CT systems. CT acquisition parameters for the first scan are available in Table 4.

**Table 4:** CT acquisition parameters used with DIQ and DMI systems

CT acquisition parameters		
	DIQ	DMI
High voltage (kV)	120	
Rotation time (s)	0.5	
Pitch	1.375	
Modulation (mA) [min; max]	Auto z [100; 300]	
Noise index (%)	18	

In addition, to minimize unnecessary radiological exposure, the second CT acquisition used a protocol with adjusted parameters for focusing specifically at attenuation correction, commonly known as CT-Based Attenuation Correction (CTAC). CTAC series were used for CT visualization during the reading session.

After anonymization, PET and CT images were then transferred to a research workstation for the ISL as described elsewhere [12]. We used a dedicated high-performance research workstation Z8 (Hewlett-Packard, Palo Alto, CA, USA) where the modelling, simulation and reconstruction were performed on a reconstruction research toolbox (Duetto v02.13, General Electric Healthcare, Chicago, IL, USA) and executed with MATLAB R2018b (The MathWorks Inc., Natick, MA, USA).

### Insertion of synthetic lesion

Synthetic lesions were inserted at the same anatomical location into the two consecutive exams of the same patient. Lesions were inserted in key organs characterized either by specific densities (lungs, bone, liver) or in the vicinity of fixed (bladder, kidney, heart) or mobile (diaphragm) organs.

We determined the same anatomical location and measured the mean activity concentration (AC) (kBq/mL) for each PET system. It was done to ensure close level of AC considering the same anatomical spot on consecutive exams of the same patient. Then, we chose the size in diameter and opted for a specific contrast, which was applied to the synthetic objects. The contrast was calculated using formula (1). We assessed the AC in the background by using a 2 cm^3^ spherical volume of interest (VOI) positioned on the insertion site prior to the simulation. We established the AC of the lesion based on the desired contrast.

1 $${\text{Contrast}}= \frac{({\text{AC}}\left({\text{Lesion}}\right)-{\text{AC}}({\text{Background}}))}{{\text{AC}}({\text{Background}})}$$

Table 5 shows the characteristics of synthetic targets in terms of shape, size, contrast, AC and anatomical location.

**Table 5:** Description of synthetic lesions

Synthetic lesions		
Number (total)	120	
Shape	Spherical	
Diameter (mm)^a^	[5; 11]	
Contrast^a^	[2; 14]	

	BMI ≤ 25	BMI > 25
Number (BMI-based)	60	60
AC (kBq/mL)^b^	6.16 [2.34; 12.55]	6.39 [3.00; 30.65]
Volume (mm^3^)^b^	484 [143; 1146]	658 [143; 1560]
Anatomical locations—number (proportion (%))		
Lungs	3 (5.0%)	6 (10.0%)
Bone	16 (26.7%)	15 (25.0%)
Liver	9 (15.0%)	6 (10.0%)
Mediastinum	16 (26.7%)	15 (25.0%)
Retroperitoneal	1 (1.6%)	0 (0.0%)
Lymph node	15 (25.0%)	18 (30.0%)

^a^[min; max]

^b^Median [min; max]

During the study, volumes, contrasts and anatomical locations of synthetic lesions were not equally distributed between the two BMI groups. The reconstructed images were then imported and stored on a dedicated interpretation workstation, the AWServer client console (General Electric Healthcare, Chicago, IL, USA), for the reading sessions using the image interpretation software PETVCAR^®^.

### Reading sessions

Two nuclear medicine physicians that were unaware of the pathological indication evaluated the images by reporting the detected lesions and then measuring the semi-quantitative values of glycolytic activity with the standardized uptake value (SUVmax, SUVmean and SUVpeak). The images were assessed in a random order, and the observers were not aware of the PET system on which the examination was performed (DMI or DIQ) or of the number of inserted synthetic lesions to be identified. The observers compared their interpretations and came to a consensus.

### Statistical analysis

The primary objective of this trial was to assess the relevance of ISL for evaluating PET performances from patient data at the lesion level. Sample size was computed for a lesion-based analysis [17, 18]. Based on the outcomes of clinical investigations, the sizes and contrasts considered for the synthetic targets and our extensive experience with these systems, we assume that the DMI should be able to detect at least 1.7 times more targets in contrast to the DIQ.

Assuming a 20% probability of agreement, 58 lesions per BMI group are required to demonstrate this difference with 80% power and a two-sided alpha of 5%. Knowing that a maximum of 15 lesions should be simulated per examination and that 58 lesions are required for each BMI group, we enrolled 12 patients per group according to the simulation strategy.

For each group of BMI (≤ 25 and > 25), lesions are simulated as follows: 4 patients without synthetic lesions (M0), 4 patients with 1 to 5 synthetic lesions (OligoM) for a total of 15 lesions and 4 patients with more than 10 synthetic lesions (MultiM) for a total of 45 lesions. The stratification of the clinical trial is illustrated in Fig. 1.

fPET/CT system and the corresponding RTPR was determined (RTPR) for synthetic lesion defined by the ratio of the detection rate of synthetic lesion by DMI and DIQ. The detection rate of synthetic lesion is defined by the ratio of the number of lesion detected by the PET and the total number of synthetic lesion. The RTPR for natural lesion was evaluated in a similar way. We assumed that the total number of natural lesions was equal to the number of natural lesions reported by at least one of the readers. Using this information, the detection rates for natural lesions were calculated for each PET/CT system and the corresponding RTPR was determined.

For assessing synthetic lesion inter-reader variability, we calculated inter-observer agreement (IOA). IOA was assessed by the concordance rate between the 2 readers. The concordance was estimated with 95% confidence interval (binomial exact). We considered an adequate inter-reader variability for IOA above 80%.

Additionally, by calculating the mean relative differences (RD) and standard deviations (SD) of lesions SUV metrics reported on the DIQ and DMI, we contrasted the semi-quantitation for synthetic and natural lesions. We determined the RD using formula (2). We calculated the SD from the distribution of the RD index.

2 $${\text{RD}}({\text{DIQ}}/{\text{DMI}})= \frac{\left({{\text{DMI}}}_{{\text{SUV}}}-{{\text{DIQ}}}_{{\text{SUV}}}\right)}{{{\text{DIQ}}}_{{\text{SUV}}}} \times 100$$

## Results

**Fig. 3:**
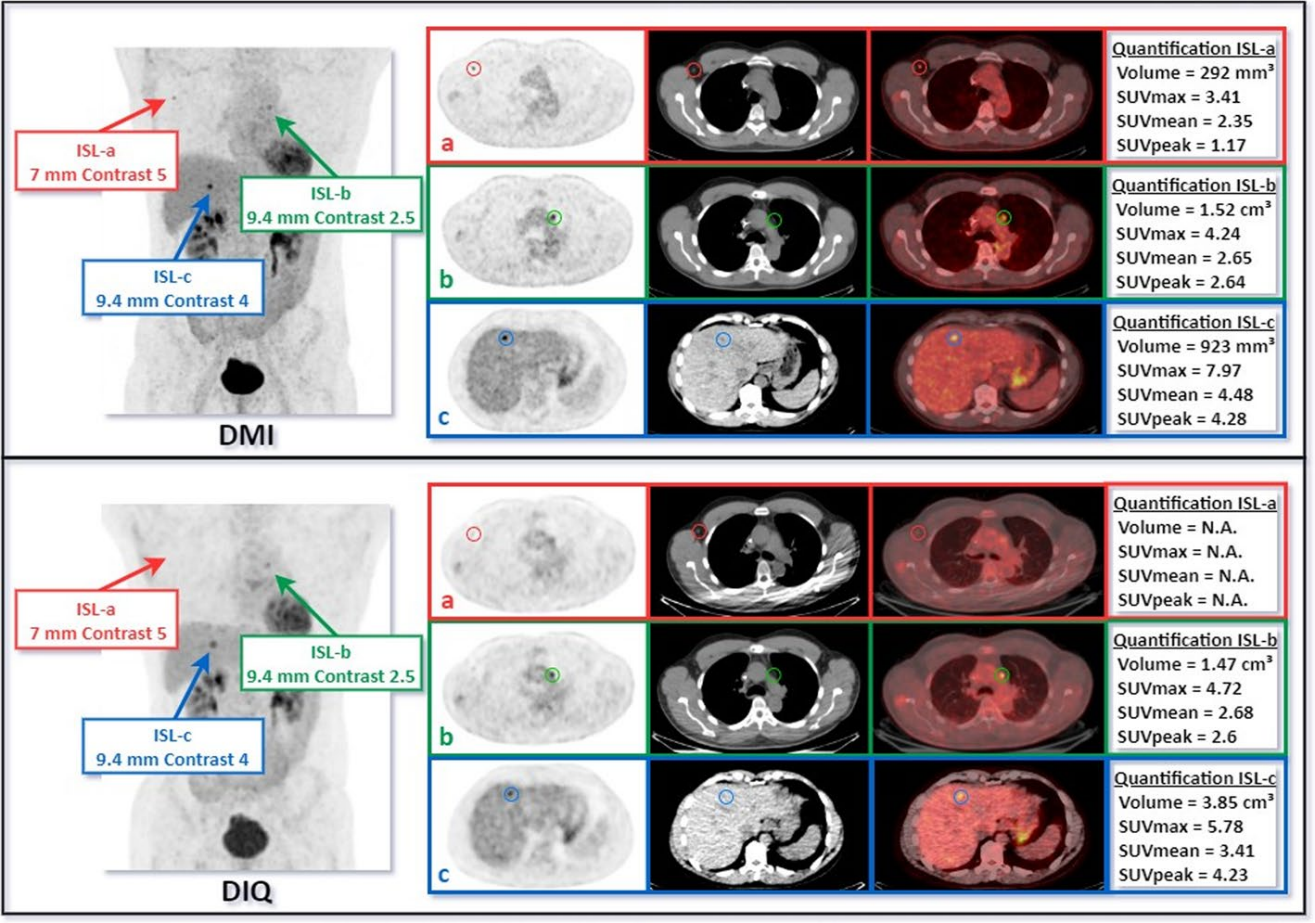
Example of ISL into two consecutive examinations of the same patient. The first examination is displayed at the top (DMI) and the second at the bottom (DIQ). Three synthetic lesions were inserted in three anatomical areas: in an axillary lymph node (a), a lesion 7 mm in diameter and a contrast of 5; in the mediastinum (b), a lesion 9.4 mm in diameter and a contrast of 2.5; in the liver (c), a lesion 9.4 mm in diameter and a contrast of 4. When reviewing the images, the physicians reported three targets on the DMI versus two on the DIQ in their consensus. For each PET/CT scanner we have from left to right: Maximum Intensity Projection (MIP), PET, CT and fused images displayed. Semi-quantitative metrics are reported in box next to axial PET, CT and fused images

We present the detection sensitivities of each system for both BMI groups. We compare the RTPR of the inserted synthetic lesions with our estimate and with the RTPR calculated from the natural lesions. We determine the inter-reader variability of synthetic lesions for the two systems considered according to the IOA. We contrast the semi-quantitative SUV metrics obtained from the lesions detected on the two consecutive examinations of the same patient. A review of a clinical case with ISL is shown in Fig. 3.

In the BMI ≤ 25 group, 59 (98.3%) and 33 (55.0%) of the 60 synthetic lesion were detected by DMI and DIQ, respectively. The relative true positive rate for synthetic lesion was 1.79 (95% Confidence Interval (CI) [1.43; 2.24]). In the BMI > 25 group, the relative true positive rate for synthetic lesion was 2.03 (95%CI [1.57; 2.64]) with 59 (98.3%) and 29 (48.3%) of the synthetic lesion detected by DMI and DIQ, respectively. 60 and 63 natural lesions were identified for ≤ 25 and > 25 BMI group, respectively. The relative true positive rates for natural lesion were 1.83 (95%CI [1.30; 2.58], DMI: 69.8%, DIQ: 38.1%) and 2.27 (95%CI [1.61; 3.21], DMI: 73.5%, DIQ: 32.4%) for ≤ 25 and > 25 BMI groups, respectively. RTPR results are summarized in Table 6.

**Table 6:** RTPR as a function of the BMI group and the PET/CT scanner

PET/CT	BMI ≤ 25 group		BMI > 25 group	
	DIQ	DMI	DMI	DIQ
Expected RTPR (DMI/DIQ)	> 1.70		> 1.70	
Natural RTPR (DMI/DIQ) 95%CI [min; max]	1.83 [1.30; 2.58]		2.27 [1.61; 3.21]	
Synthetic RTPR (DMI/DIQ) 95%CI [min; max]	1.79 [1.43; 2.24]		2.03 [1.57; 2.64]	

In the BMI ≤ 25 group, IOA was 88.3% (95%CI [77.4; 95.2]) and 86.7% (95%CI [75.4; 94.1]) for DMI and DIQ, respectively. In the BMI > 25 group, IOA was 96.7% (95%CI [88.5; 99.6]) and 93.3% (95%CI [83.8; 98.2]) for DMI and DIQ, respectively. In both cases, we checked the condition of an IOA above 80%.

Table 7 shows RD and SD between DMI and DIQ in terms of semi-quantitative metrics (SUVmax, SUVmean and SUVpeak) for natural and synthetic lesions and each group of BMI. N represents the number of lesions detected simultaneously on both scanners.

**Table 7:** Differences in semi-quantitative metrics for natural and synthetic lesions

	Synthetic		Natural	
	BMI ≤ 25 group (*N* = 33)	BMI > 25 group (*N* = 29)	BMI ≤ 25 group (*N* = 18)	BMI > 25 group (*N* = 19)
SUVmax				
RD ± SD	58.7 ± 58.9	68.8 ± 60.0	54.5 ± 54.2	69.4 ± 48.2
Median [min; max]	52.9 [− 27.5; 229.7]	61.9 [− 10.7; 278.3]	43.5 [− 14.6; 163.9]	68.5 [− 13.3; 183.3]
SUVmean				
RD ± SD	67.3 ± 67.1	68.7 ± 60.5	59.3 ± 57.5	68.6 ± 44.6
Median [min; max]	61.0 [− 31.3; 258.3]	67.5 [− 6.0; 264.3]	44.9 [− 13.9; 184.9]	81.4 [− 11.5; 146.9]
SUVpeak				
RD ± SD	15.4 ± 28.0	18.3 ± 22.6	13.7 ± 20.0	22.3 ± 19.1
Median [min; max]	9.1 [− 23.8; 105.0]	20.5 [− 16.0; 57.6]	9.4 [− 12.9; 50.6]	27.8 [− 10.9; 51.0]

## Discussion

The performance of PET scanners can be evaluated in several ways but each has its drawbacks. Phantom studies are relevant for investigating and benchmarking scanners using a standard object whose parameters are controlled, but they lack realism in comparison with patient anatomy [7, 8]. On the other hand, clinical studies evaluate performances directly on patient exams but suffer from a lack of ground truth [19]. Some clinical studies have used a scan-rescan imaging protocol of the same patient to compare datasets acquired from two different PET systems. By doing so, it is possible to establish differences between two systems and to compare them [20–22]. However, as with classical clinical studies, there is still no ground truth, so it is not possible to conclude whether one device outperforms the other.

In this study, we simulate lesions with controlled and well-defined characteristics that we deemed pertinent for evaluating the detection rate of current PET/CT scanners. The goal was to use ISL in a clinical study to establish the detection rate from exams of the same patient acquired on different PET scanners in a scan-rescan process and therefore evaluate its relevance to compare clinical performances of PET systems.

We applied the simulation method on data from real anatomical models, thereby approaching clinical practice more accurately. We performed simulation to target and control synthetic data, and thus providing a reliable alternative to conventional invasive methods for establishing the ground truth on clinical data. We used the same acquisition and reconstruction parameters applied in clinical practice in our department. We aimed to compare the performance of each system under our real clinical conditions.

The RTPR for synthetic lesions was consistent with our initial estimate and with the RTPR measured on natural lesions. In addition, inter-observer agreement (IOA) is high and above 85% for all scanners and BMI groups. As shown in Table 7, we observe differences between the two scanners consistent contrasting synthetic and natural lesions for all SUV metrics described. We report strictly positive RD values, resulting in higher semi-quantitative metrics for DMI when compared to DIQ. Additionally, we report a trend related to the BMI group. Indeed, a slight shift is observed and is consistent for synthetic and natural lesions. It is consistent with the influence of BMI on PET detection rate and semi-quantitation [23, 24].

We observe satisfactory results in terms of the detection rates of synthetic lesions compared to our initial estimate and the values found for natural lesions. The agreement among observers regarding the synthetic lesions has been deemed convincing. The semi-quantitative metrics have demonstrated consistent and close results between natural and synthetic lesions. The method generates synthetic data equivalent to pathological lesions in terms of realism and clinical context and is consistent with measurements made on natural lesions. These findings confirm our objective of demonstrating the relevance of ISL applied on patient data to evaluate PET performances in a clinical manner. Ancillary, we did not identify specific thresholds for activity concentration or volume that would lead to undetected targets.

To our knowledge, no clinical study has attempted to implement such a simulation method in a clinical trial using a design based on two consecutive scans of the same patient. By doing so, we added synthetic lesions at the same location on two consecutive examinations in the same patient in order to create a single clinical case resulting from each of the PET scanners as depicted in Fig. 3. Thus, the insertion of synthetic lesions in realistic images helps to evaluate, compare and optimize the performance of these PET systems.

In a scan-rescan process, a significant bias may arise from the uptake time of the radiolabelled product, which may be slightly longer for the second acquisition. The delay in our study was around 25 min. The 25-min interval includes an unavoidable delay, which comprises the standard duration of the first PET acquisition (10 min). Subsequently, the patient relocates to another examination room for the second acquisition, which takes around 15 min. Hence, the distribution of the product in the patient’s body may vary owing to the interval between the two examinations. We know that the activity present within the patient over time is not strictly fixed and stable. For example, some lesions have a greater perfusion of radiotracer when the uptake time in tissues increases. To avoid the second acquisition to be performed systematically on the same system and thereby creating a potential bias in the detection and semi-quantitation of lesions, we balanced the order of scan performed on each system (12 on the DMI then the DIQ and 12 on the DIQ then the DMI).

Furthermore, we performed time resampling on PET data using list-mode to address another limitation, the radioactive decay. Given that the second acquisition began roughly 25 min after the first one, it was extended for several minutes to compensate for the radioactive decay of 18F-FDG. The raw data were adjusted to match the total counts obtained during the first acquisition, allowing a robust comparison of the two examinations with similar statistics (less than a 1% difference).

Additionally, since we determine spatial coordinates for each insertion site from the CT images. A mismatch between PET and CT could have a direct impact on the synthetic lesion positioning. Hence, with the current proposed method, it could generate discrepancies between the consecutive examinations of the same patient. In this study, at the end of each acquisition, the technologists ensured that the PET and CT images were correctly superposed and correlated.

The radiological exposure associated with the second CT acquisition has been reduced by modifying the acquisition and reconstruction parameters. To ensure that physicians do not interpret examinations with variable CT image quality, we standardized the entire dataset using the same CT series for visualization typically employed for attenuation correction: the CTAC.

## Conclusion

ISL can be used to evaluate performance differences between PET/CT scanners. Using synthetically modified clinical images, it is possible to produce a controlled ground truth in a realistic anatomical model and thus evaluate and optimize the potential of PET/CT scanners in clinical settings. It is therefore an assumption that this method could be of great interest for the evaluation of artificial intelligence (AI)-based reconstruction algorithms.

## Data Availability

The datasets used and/or analysed during the current study are available from the corresponding author on reasonable request. The data are not publicly available due to ethical restriction.

## Abbreviations

^18^F-FDG: 2-Deoxy-2-[18F]fluoroglucose
AI: Artificial intelligence
AC: Activity concentration
BGO: Bismuth germanate
BMI: Body mass index
BPL: Bayesian penalized likelihood
CI: Confidence interval
CT: Computed tomography
CTAC: Computer tomography-based attenuation correction
DIQ: Discovery-IQ
DMI: Discovery-MI
EANM: European Association of Nuclear Medicine
EARL: EANM research GmbH
FOV: Field of view
IEC: International Electrotechnical Commission
IOA: Inter-observer agreement
ISL: Insertion of synthetic lesion
IUCT-O: Institut Universitaire du Cancer de Toulouse—Oncopole
LYSO: Lutetium–yttrium-oxyorthosilicate
M0: No inserted lesions
MultiM: 10 to 15 inserted lesions
NEMA: National Electrical Manufacturers Association
OligoM: 1 to 5 inserted lesions
PET: Positron emission tomography
PSF: Point spread function
RD: Relative difference
RTPR: Relative true positive rate
SD: Standard deviation
SUV: Standardized uptake value
TOF: Time of flight
VOI: Volume of interest
